# Somatic mutation of *PTEN* in bladder carcinoma

**DOI:** 10.1038/sj.bjc.6690439

**Published:** 1999-05

**Authors:** J S Aveyard, A Skilleter, T Habuchi, M A Knowles

**Affiliations:** ICRF Cancer Medicine Research Unit, St James's University Hospital, Beckett Street, Leeds, LS9 7TF, UK; Department of Urology, Graduate School of Medicine, Kyoto University, Sakyo-ku, Kyoto, 606, Japan

**Keywords:** *PTEN*, transitional cell carcinoma, bladder, chromosome 10, loss of heterozygosity

## Abstract

The tumour suppressor gene *PTEN/MMAC1*, which is mutated or homozygously deleted in glioma, breast and prostate cancer, is mapped to a region of 10q which shows loss of heterozygosity (LOH) in bladder cancer. We screened 123 bladder tumours for LOH in the region of *PTEN*. In 53 informative muscle invasive tumours (≥ pT2), allele loss was detected in 13 (24.5%) and allelic imbalance in four tumours (overall frequency 32%). LOH was found in four of 60 (6.6%) informative, non-invasive tumours (pTa/pT1). We screened 63 muscle invasive tumours for *PTEN* mutations by single-strand conformation polymorphism (SSCP) analysis and for homozygous deletion by duplex quantitative polymerase chain reaction (PCR). Two homozygous deletions were identified but no mutations. Of 15 bladder tumour cell lines analysed, three showed homozygous deletion of all or part of the *PTEN* gene, but none had mutations detectable by SSCP analysis. Our results indicate that *PTEN* is involved in the development of some bladder tumours. The low frequency of mutation of the retained allele in tumours with 10q23 LOH suggests that there may be another predominant mechanism of inactivation of the second allele, for example small intragenic deletions, that hemizygosity may be sufficient for phenotypic effect, or that there is another target gene at 10q23. © 1999 Cancer Research Campaign


British Journal of Cancer (1999) 80(5/6), 904–908
© 1999 Cancer Research Campaign
Article no. bjoc.1998.0439

### Somatic mutation of *PTEN* in bladder carcinoma

**JS Aveyard[1], A Skilleter[1], T Habuchi[2] and MA Knowles[1]**

[1]ICRF Cancer Medicine Research Unit, St James's University Hospital, Beckett Street, Leeds LS9 7TF, UK; [2]Department of Urology, Graduate School of Medicine, Kyoto University, 54 Kawahara-cho, Sakyo-ku, Kyoto 606, Japan

**Summary** The tumour suppressor gene *PTEN/MMAC1*, which is mutated or homozygously deleted in glioma, breast and prostate cancer, is mapped to a region of 10q which shows loss of heterozygosity (LOH) in bladder cancer. We screened 123 bladder tumours for LOH in the region of *PTEN*. In 53 informative muscle invasive tumours (≥ pT2), allele loss was detected in 13 (24.5%) and allelic imbalance in four tumours (overall frequency 32%). LOH was found in four of 60 (6.6%) informative, non-invasive tumours (pTa/pT1). We screened 63 muscle invasive tumours for *PTEN* mutations by single-strand conformation polymorphism (SSCP) analysis and for homozygous deletion by duplex quantitative polymerase chain reaction (PCR). Two homozygous deletions were identified but no mutations. Of 15 bladder tumour cell lines analysed, three showed homozygous deletion of all or part of the *PTEN* gene, but none had mutations detectable by SSCP analysis. Our results indicate that *PTEN* is involved in the development of some bladder tumours. The low frequency of mutation of the retained allele in tumours with 10q23 LOH suggests that there may be another predominant mechanism of inactivation of the second allele, for example small intragenic deletions, that hemizygosity may be sufficient for phenotypic effect, or that there is another target gene at 10q23.

**Keywords:** *PTEN*; transitional cell carcinoma; bladder; chromosome 10; loss of heterozygosity

Deletions of the long arm of chromosome 10 have been described in many tumour types, including carcinoma of the prostate, uterus, glioblastoma, meningioma and melanoma (Rasheed et al, 1992; Isshiki et al, 1993; Rempel et al, 1993; Jones et al, 1994; Gray et al, 1995; Cairns et al, 1997). Recently, loss of heterozygosity (LOH) of 10q has been reported in bladder tumours (Cappellen et al, 1997; Kagan et al, 1998), confirming previous cytogenetic reports of monosomy 10 and 10q deletion (Gibas et al, 1984; Atkin and Baker, 1985; Berger et al, 1986; Babu et al, 1987; Smeets et al, 1987) and comparative genomic hybridization and fluorescence in situ hybridization studies (Kallioniemi et al, 1992; Wang et al, 1994). A critical region of deletion defined in these studies is coincident with the major region mapped in other tumour types. Recently, the *PTEN*/*MMAC1* gene was identified as a candidate tumour suppressor gene within this region at 10q23 (Li et al, 1997; Steck et al, 1997). Mutations of *PTEN* have been described in glioblastomas (Rasheed et al, 1992; Wang et al, 1994; Cairns et al, 1997; Steck et al, 1997), carcinomas of the prostate (Steck et al, 1997) and breast (Rhei et al, 1997), endometrial carcinoma (Risinger et al, 1997) and melanoma (Guldberg et al, 1997), indicating that *PTEN* is the likely target of deletions in these cases. Recently, a low frequency of *PTEN* mutation has been reported in bladder cancer (Cairns et al, 1998). In addition, germline mutations of PTEN are present in the familial cancer predisposition syndromes Cowden disease, Lhermite–Duclos disease and Bannayan–Zonana syndrome (Liaw et al, 1997; Marsh et al, 1997; Nelen et al, 1997).

Transitional cell carcinomas (TCC) of the bladder constitute two distinct clinical phenotypes. The majority of tumours at presentation (~80%) are low grade non-invasive lesions which may recur locally but progress infrequently. In contrast, those tumours which are muscle invasive at presentation (~20%) often progress rapidly and have poor prognosis. Previous molecular genetic analyses have identified LOH on several chromosome arms including 3p, 4p, 4q, 8p, 9p, 9q, 11p, 13q, 14q, 17p and 18q in TCC (Presti et al, 1991; Brewster et al, 1994; Knowles et al, 1994; Chang et al, 1995; Polascik et al, 1995). Apart from alterations to chromosome 9, all of these changes are more common in muscle-invasive tumours and to date none, assessed individually, are good prognostic markers either for superficial papillary tumours or for tumours that are locally invasive at diagnosis. LOH on 10q has been reported to be more frequent in muscle-invasive TCC (Cappellen et al, 1997). The purpose of this study was to assess the frequency of LOH at 10q23 and to determine the frequency of mutation of *PTEN* in a series of non-invasive (Ta/T1) and muscle-invasive TCCs.

*Received 16 September 1998*
*Revised 12 November 1998*
*Accepted 24 November 1998*

*Correspondence to:* MA Knowles

### MATERIALS AND METHODS

### Tumours and cell lines

Tumour samples were obtained from patients undergoing endoscopic resection of bladder carcinoma. Tissues were frozen immediately at –20°C or in liquid nitrogen and stored at –70°C. Adjacent portions of each tumour were fixed for histopathological examination. Tumour grade and stage were assessed according to the TNM (tumour, node, metastasis) system (UICC, 1978). Tissues were thawed, dissected and DNA was extracted as described (Proctor et al, 1991). Some of these DNA samples have been used in previous published studies of LOH and mutation analyses in bladder cancer. All have been assessed for LOH at multiple loci and are believed to contain at least 70% tumour cells based on these analyses. A venous blood sample was obtained from each patient as a source of constitutional DNA, and leucocyte DNA was extracted (Proctor et al, 1991). The cell lines used were RT112, RT4, T24, HT1376, HT1197, 253J, 5637, SD, J'ON, UM-UC-3, VM-CUB-II, SCaBER, J82, SW1710 and 609 (Knowles, 1992).

### LOH analysis

Three highly polymorphic microsatellite markers, *D10S541*, *D10S1765* and *D10S215*, were used to assess LOH at 10q23. These markers map close to *PTEN* based on physical maps of the region. Forward primers were labelled with $^{32}$P using [$\gamma^{32}$P] adenosine 5′-triphosphate (ATP) and T4 polynucleotide kinase. Polymerase chain reactions (PCRs) were carried out in 1 × PCR buffer (Perkin-Elmer Corp) with 200 μM deoxynucleotide triphosphates, 2 pmol of each primer and 0.125 units of *Taq* DNA polymerase in a total volume of 12.5 μl. Twenty-seven cycles of 95°C for 1 min, 55°C for 1 min and 72°C for 1.5 min were carried out followed by an extension at 72°C for 10 min. A total of 7.5 μl loading buffer (95% formamide, 10 mM EDTA, 0.02% bromophenol blue and 0.02% xylene cyanol) was added, samples were denatured at 94°C for 5 min and loaded onto 6% denaturing polyacrylamide gels (National Diagnostics). Following electrophoresis, gels were dried and exposed to Fuji RX film for 1–6 h. LOH was assessed by eye and only cases with clear reduction in signal from one allele (> 50%) were scored. 'Partial' loss of an allele or 'allelic imbalance' was also recorded.

### Single-strand conformation polymorphism analysis

Single-strand conformational polymorphism (SSCP) analysis was carried out as described (Knowles and Williamson, 1993). Gels consisted of 0.5X mutation detection enhancement solution (MDE) (FMC BioProducts) and were run at 6–8 watts for 16–18 h at room temperature. Bands with altered mobility were excised and eluted from the gels and amplified by PCR using the initial PCR primers. PCR fragments were isolated by agarose gel electrophoresis and purified for sequencing using the QIAquick Gel Extraction Kit (Qiagen). Sequencing reactions were carried out using the ABI PRISM Dye Terminator Cycle Sequencing Kit (Perkin-Elmer) and the products were analysed on an ABI 377 DNA Sequencer. Sequencing of both strands was carried out using the initial PCR primers.

### Homozygous deletion analysis

Cell line DNAs were used as templates in duplex PCR reactions with primers for a 300 bp fragment of the enolase gene (*ENO*, chromosome 12p) as control (forward primer: TGTGATTCC-CTCTTTGCTTGCC; reverse primer: TGATCATTCTTCCACG-GACC). Primer sequences for *PTEN* are shown in Table 1. PCR reactions were as for microsatellite analysis but for 30 cycles. Products were run in 1% agarose gels. DNA from primary tumours was amplified in duplex reactions containing primers for a microsatellite marker at which LOH had been detected and primers for either *D10S2491*, a CA repeat polymorphism within *PTEN* (Cairns et al, 1997), or primers for *PTEN* exon 8 (8/1F, 8/1R, Table 1). Reactions were as for microsatellite PCR but with 23 cycles. Products were run in 6% denaturing polyacrylamide gels.

**Table 1** Primers used for SSCP analysis

| Exon | Primer | Sequence | Product size (bp) |
|---|---|---|---|
| 1 | PTEN1F | AGTCGCTGCAACCATCCA | |
| | PTEN1R | TCTAAGAGAGTGACAGAAAGGTA | 319 |
| 2 | PTEN2F | CTGCATATTTCAGATATTTCTTTCC | |
| | PTEN2R | CTGTGGCTTAGAAATCTTTTC | 214 |
| 3 | PTEN3F | CCGTTCGTACGAGAATCGCT | |
| | PTEN3R | TCTACCTCACTCTAACAAGC | 212 |
| 4 | PTEN4F | GGCAATGTTTGTTAGTATTAGTAC | |
| | PTEN4R | TCGGGTTTAAGTTATACAACATAG | 171 |
| 5 | PTEN5/1F | GCAACATTTCTAAAGTTACCTACTTG | |
| | PTEN5/1R | CATATCATTACACCAGTTCG | 236 |
| | PTEN5/2F | GACAATCATGTTGCAGCAAT | |
| | PTEN5/2R | CCAATAAATTCTCAGATCCAGG | 202 |
| 6 | PTEN6F | TACGACCCAGTTACCATAGC | |
| | PTEN6R | GAAGGATGAGATTCAAGCAC | 254 |
| 7 | PTEN7F | TGGTATGTATTTAACCATGC | |
| | PTEN7R | CCTTATTTTGGATATTTCTCCC | 230 |
| 8 | PTEN8/1F | TGCAAATGTTTAACATAGGTGA | |
| | PTEN8/1R | CCTTGTCATTATCTGCACGC | 244 |
| | PTEN8/2F | GGAAGTCTATGTGATCAAGA | |
| | PTEN8/2R | CGTAAACACTGCTTCGAAATA | 285 |
| 9 | PTEN9/1F | AGATGAGTCATATTTGTGGG | |
| | PTEN9/1R | CTAACATCTGGTGTTACAGA | 128 |
| | PTEN9/2F | CGTCAAATCCAGAGGCTAG | |
| | PTEN9/2R | TCATGGTGTTTTATCCCTCTTG | 186 |

### Reverse transcriptase PCR analysis

Total RNA was extracted from cultured cells using RNAzol (Biogenesis, UK). First-strand cDNA was synthesized using the Clontech Advantage RT-for-PCR Kit and oligo-dT as primer. Two pairs of primers were designed to amplify the 5′ and 3′ regions of the cDNA individually. A 5′ end fragment of 280-bp was amplified using a forward primer in exon 1 (CATCATCAAAGAGAT-CGTTAGC) and a reverse primer in exon 5 (TGGGTTA-TGGTCTTCAAAAGG). A 3′ fragment of 150-bp was amplified using a forward primer in exon 8 (GCGTGCAGATAATGA-CAAGG) and a reverse primer in exon 9(CCTCTGGATT-TGACGGCTCC). These sequences are conserved in the *PTEN* pseudogene (Dahia et al, 1998). cDNA preparations were done in the presence and absence of reverse transcriptase, the latter acting as a control for contaminating genomic DNA from which fragments of the pseudogene can be amplified with these primers. No products were seen in these controls indicating minimal contamination with genomic DNA. Genomic fragments of *PTEN* are too large to be amplified by PCR under the conditions used which consisted of 30 cycles as described above for microsatellite analysis.

### RESULTS

### LOH and homozygous deletion at 10q23

A total of 123 bladder tumour samples were analysed for LOH in the region of the *PTEN* gene. These comprised 58 muscle invasive tumours and 65 non-invasive tumours classified as stage Ta or T1 (UICC, 1978). Three markers, *D10S541*, *D10S1765* and *D10S215*, which closely flank *PTEN*, were used. All but ten tumours (five invasive, five superficial) were informative for at least one marker. Allelic loss was detected in 13 muscle-invasive tumours (24.5%) and four superficial tumours (6.6%). An additional four

© Cancer Research Campaign 1999 

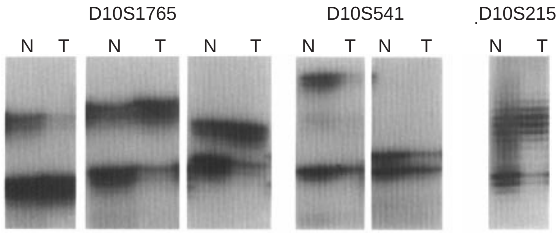

**Figure 1** Examples of LOH at three 10q23 loci in six bladder tumours. N, leucocyte DNA template; T, tumour DNA template

**Table 2** Loss of heterozygosity at 10q23 in transitional cell carcinoma

| Marker | Frequency of LOH (LOH/informative cases) | Overall frequency |
|---|---|---|
| Superficial tumours (Ta/T1) | | |
| D10S541 | 1/33 | |
| D10S1765 | 3/65 | 4/60 (6.6%) |
| Invasive tumours (>T2) | | |
| D10S215 | 5/20 | |
| D10S541 | 6/23 | 13/53 LOH<br>4/53 allelic imbalance |
| D10S1765 | 8/27 | Total 17/53 (32%) |

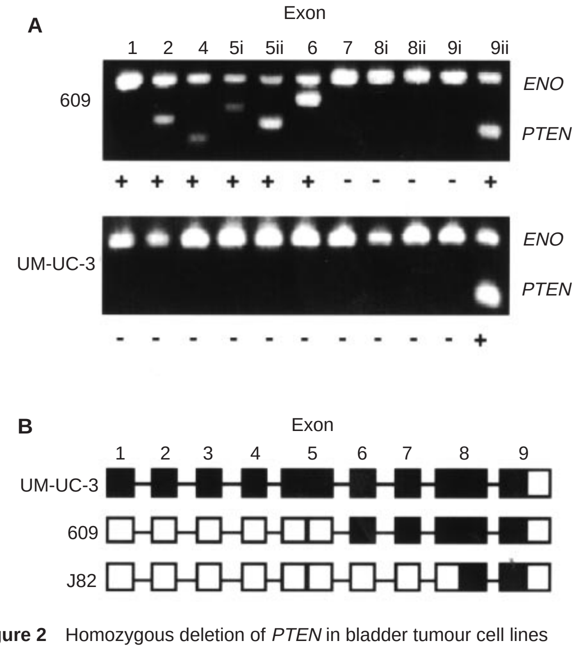

**Figure 2** Homozygous deletion of *PTEN* in bladder tumour cell lines identified by duplex PCR. (**A**) Examples of duplex PCR products from the cell lines UM-UC-3 and 609 with primers for *PTEN* exons and a 300 bp fragment of the enolase gene (*ENO*). Exon 1 is seen as a doublet with *ENO* in the 609 sample but is absent from UM-UC-3. (**B**) Map of homozygous deletions identified. ■ Homozygous deletion, □ Retention muscle-invasive tumours showed allelic imbalance, which may indicate either underrepresentation or low level amplification of one allele. The overall frequency of LOH/allelic imbalance at 10q23 in the muscle-invasive TCCs was, therefore, 32% (Table 2). Examples are shown in Figure 1.

We also studied a series of 15 bladder cancer cell lines. Constitutional DNA was available for one of the patients from whom a cell line was established (J82). This cell line showed LOH in the *PTEN* region (data not shown). Since LOH analysis of the other cell lines was not possible, we carried out duplex PCR analysis to screen for possible homozygous deletion of *PTEN*. PCR was carried out using primers for each exon of *PTEN* and a fragment of the enolase gene (*ENO*) as control. The cell line UM-UC-3 showed homozygous deletion of all exons of the gene apart from the 3′ fragment of exon 9 (9/2 Table 1). Homozygous deletions were also identified in J82 and 609 (Figure 2). Since the primers for the 3′ fragment of exon 9 were located within the *PTEN* cDNA sequence, the products obtained from these cell lines may be derived from the *PTEN* pseudogene. The 3′ limit of deletion therefore remains to be determined.

To identify possible homozygous deletions of *PTEN* in tumours with 10q23 LOH, we carried out duplex PCR on all tumours with LOH using primers for a CA repeat marker within the *PTEN* gene (*D10S2491*) and primers for a locus at which LOH had been identified. Using this assay, homozygous deletion of *PTEN* is indicated if apparent retention of heterozygosity is detected within the gene in tumours, with LOH of flanking markers. Homozygous deletion was detected in one tumour and all other informative tumours showed clear LOH at *D10S2491*. Since two of the three cell lines with homozygous deletion had shown deletion of the 3′ region of the gene, we then carried out duplex PCR on seven tumours with clear LOH at *D10S2491* and primers for part of *PTEN* exon 8. One more intragenic homozygous deletion was detected by this method.

### SSCP and sequence analysis

Intronic primers were designed to amplify the entire coding region of PTEN in 11 fragments containing all splice junctions (Table 1) in 63 muscle-invasive tumour samples, including all cases with LOH/allelic imbalance. Bands with altered mobility were detected in 13 cases. Altered bands were excised from SSCP gels, re-amplified and sequenced. Three of these, which were in exon 1, were also detected in the constitutional DNA of the individual concerned and were considered likely to be normal sequence polymorphisms. Sequencing analysis showed this to be a single base change (–9C/G). No tumour-specific mutations were detected.

### RT-PCR analysis

Seven bladder cell lines (EJ, J82, RT112, RT4, SD, UM-UC-3 and 609) were examined by RT-PCR for expression of *PTEN* mRNA. Primers were designed to amplify fragments from the 5′ and 3′ ends of the RNA. Results were compatible with the extent of homozygous deletion identified in the gene. Cell lines with homozygous deletion showed absence of one or both products and all other cell lines showed products of the expected size from both ends of the transcript.

### DISCUSSION

We have shown that LOH at 10q23 is found predominantly in muscle invasive bladder tumours. Our finding of LOH or allelic imbalance in 32% of tumours ≥ pT2 and in only 6.6% of superficial (Ta/T1) tumours confirms that of Cappellen et al (1997). This marked tumour stage-associated difference is the likely

© Cancer Research Campaign 1999

explanation for the low overall frequencies of 10q deletion found in some previous studies which examined predominantly superficial tumours (Knowles et al, 1994; Kallioniemi et al, 1995).

A recent study by Kagan et al (1998) pinpointed a critical region of LOH at 10q23 in bladder cancer between the loci *D10S1644* and *D10S541*, an interval that contains the *PTEN* gene. All of the tumours studied were grade III invasive tumours, 9/20 of which had discrete deletions focused on this region. *PTEN* is therefore a good candidate gene for the target of these deletions in bladder cancer.

We found no tumour-specific mutations by SSCP analysis in the panel of 63 muscle-invasive tumours analysed, which included 17 with allelic imbalance at 10q23. Although SSCP does not detect all mutations, small deletions and insertions and most point mutations should be identified. Indeed, we have identified 18 of 20 known small sequence alterations, including seven point mutations and 11 single or dinucleotide insertion/deletion mutations in DNA samples from familial cases of tuberous sclerosis (TS) for which the inactivation was known (DNA kindly provided by Professor Sue Povey) using the SSCP conditions described here (Hornigold et al, unpublished results). An advantage of SSCP analysis on DNA derived from tumour tissues is that mutant alleles can be identified even in the presence of significant normal tissue contamination. However, stromal contamination was not a problem in these samples since LOH was easy to score. Only four cases showed allelic imbalance rather than clear LOH, which may indicate higher levels of stromal contamination in a few cases. The recent report by Cairns et al (1998) that identified only two mutations in a panel of 25 tumours with 10q LOH is in accord with this. We conclude that small sequence variants of *PTEN* are rare in bladder cancer.

The finding of three homozygous deletions of all or part of *PTEN* in cell lines and of two homozygous deletions in tumours suggests that this is a more common mechanism of inactivation. Similarly, Cairns et al (1998) found four apparent homozygous deletions in a series of 65 bladder tumours with 10q LOH, and Teng et al (1997) have reported homozygous deletion of the entire gene in a bladder tumour cell line. The finding of more frequent homozygous deletion than small sequence alterations in the retained allele is reminiscent of the situation for the 9p21 tumour suppressor gene *CDKN2*/p16 in which point mutations are infrequent but homozygous deletion is more common in bladder cancer. The type of mutation found may reflect the nature of the inducing mutagen(s) in a particular tissue or result from other genetic changes in the tumour concerned. For example, *PTEN* point mutations are common in endometrial cancers and particularly those tumours which show microsatellite instability and may generate single base substitutions at high frequency (Kong et al, 1997).

The frequencies of homozygous deletion and mutation found in the tumour samples were much lower than the frequency of LOH in the region. Since we did not map the region of LOH in detail in these tumours, it is possible that LOH spanned a large part of the chromosome and the target of this may be another chromosome 10 gene in some cases. The recent results of Kagan et al (1998) suggest that this is unlikely since most of the deletions they identified were clustered around *PTEN*. Fine mapping in the region of *PTEN* may clarify this. Our finding of intragenic deletions of *PTEN* in cell lines, however, points to this gene as the likely target. The two small intragenic homozygous deletions in cell lines would not have been identified using the marker *D10S2741*, which was used for the tumour specimens. Since we were able only to use a non-polymorphic intragenic sequence to examine tumour specimens in the small region deleted in the cell lines, it is possible that some homozygous deletions were missed in the tumours. It is likely, therefore, that our finding of only two tumours with homozygous deletion is an underestimate of the true frequency. Only accurate assessment of homozygous deletion using carefully microdissected tumour tissues and multiple intragenic markers will clarify this.

An alternative explanation for the discrepancy between LOH frequency and involvement of *PTEN* is that loss of function of a single allele may be sufficient to generate an altered phenotype. Loss of the second allele may contribute at a later step in tumour progression and, in the case of cell lines, may be selected for during in vitro establishment. Certainly, several studies of *PTEN* have failed to find mutations at the expected frequency, particularly those on prostate and thyroid tumours (Cairns et al, 1997; Dahia et al, 1997; Pesche et al, 1998).

Finally, recent results that confirm a low frequency of homozygous deletion and mutation in prostate tumour xenografts (Whang et al, 1998) suggest that *PTEN* may be transcriptionally silenced in some cases by hypermethylation. Examination of the methylation status of the retained *PTEN* allele in bladder tumours with LOH must now be carried out.

### ACKNOWLEDGEMENTS

This work was funded by Marie Curie Cancer Care and The Imperial Cancer Research Fund.

### REFERENCES

Atkin NB and Baker MC (1985) Cytogenetic study of ten carcinomas of the bladder: involvement of. *Cancer Genet Cytogenet* **15**: 253–268

Babu VR, Lutz MD, Miles BJ, Farah RN, Weiss L and Van Dyke DL (1987) Tumor behavior in transitional cell carcinoma of the bladder in relation to chromosomal markers and histopathology. *Cancer Res* **47**: 6800–6805

Berger CS, Sandberg AA, Todd IA, Pennington RD, Haddad FS, Hecht BK and Hecht F (1986) Chromosomes in kidney, ureter, and bladder cancer. *Cancer Genet Cytogenet* **23**: 1–24

Brewster SF, Gingell JC, Browne S and Brown KW (1994) Loss of heterozygosity on chromosome 18q is associated with muscle-invasive transitional cell carcinoma of the bladder. *Br J Cancer* **70**: 697–700

Cairns P, Okami K, Halachmi S, Halachmi N, Esteller M, Herman JG, Jen J, Isaacs WB, Bova GS and Sidransky D (1997) Frequent inactivation of PTEN/MMAC1 in primary prostate cancer. *Cancer Res* **57**: 4997–5000

Cairns P, Evron E, Okami K, Halachmi N, Esteller M, Herman JG, Bose S, Wang SI, Parsons R and Sidransky D (1998) Point mutation and homozygous deletion of PTEN/MMAC1 in primary bladder cancers. *Oncogene* **16**: 3215–3218

Cappellen D, Gil Diez de Medina S, Chopin D, Thiery JP and Radvanyi F (1997) Frequent loss of heterozygosity on chromosome 10q in muscle-invasive transitional cell carcinomas of the bladder. *Oncogene* **14**: 3059–3066

Chang WY-H, Cairns P, Schoenberg MP, Polascik TJ and Sidransky D (1995) Novel suppressor loci on chromosome 14q in primary bladder cancer. *Cancer Res* **55**: 3246–3249

Dahia PL, Marsh DJ, Zheng Z, Zedenius J, Komminoth P, Frisk T, Wallin G, Parsons R, Longy M, Larsson C and Eng C (1997) Somatic deletions and mutations in the Cowden disease gene, PTEN, in sporadic thyroid tumors. *Cancer Res* **57**: 4710–4713

Dahia PL, FitzGerald MG, Zhang X, Marsh DJ, Zheng Z, Pietsch T, von Deimling A, Haluska FG, Haber DA and Eng C (1998) A highly conserved processed PTEN pseudogene is located on chromosome band 9p21. *Oncogene* **16**: 2403–2406

Gibas Z, Prout GR Jr, Connolly JG, Pontes JE and Sandberg AA (1984) Nonrandom chromosomal changes in transitional cell carcinoma of the bladder. *Cancer Res* **44**: 1257–1264

© Cancer Research Campaign 1999 

Gray IC, Phillips SM, Lee SJ, Neoptolemos JP, Weissenbach J and Spurr NK (1995) Loss of the chromosomal region 10q23–25 in prostate cancer. *Cancer Res* **55**: 4800–4803

Guldberg P, thor Straten P, Birck A, Ahrenkiel V, Kirkin AF and Zeuthen J (1997) Disruption of the MMAC1/PTEN gene by deletion or mutation is a frequent event in malignant melanoma. *Cancer Res* **57**: 3660–3663

Isshiki K, Elder DE, Guerry D and Linnenbach AJ (1993) Chromosome 10 allelic loss in malignant melanoma. *Genes Chromosomes Cancer* **8**: 178–184

Jones MH, Koi S, Fujimoto I, Hasumi K, Kato K and Nakamura Y (1994) Allelotype of uterine cancer by analysis of RFLP and microsatellite. *Genes Chromosomes Cancer* **9**: 119–123

Kagan J, Liu J, Stein JD, Wagner SS, Babkowski R, Grossman BH and Katz RL (1998) Cluster of allele losses within a 2.5 cM region of chromosome 10 in high-grade invasive bladder cancer. *Oncogene* **16**: 909–913

Kallioniemi A, Kallioniemi O-P, Sudar D, Rutovitz D, Gray JW, Waldman FM and Pinkel D (1992) Comparative genomic hybridization for molecular genetic analysis of solid tumors. *Science* **258**: 818–821

Kallioniemi A, Kallioniemi O-P, Citro G, Sauter G, DeVries S, Kerschmann R, Caroll P and Waldman F (1995) Identification of gains and losses of DNA sequences in primary bladder cancer by comparative genomic hybridisation. *Genes Chromosomes Cancer* **12**: 213–219

Knowles M (1992) Human papillomavirus sequences are not detectable by Southern blotting or general primer-mediated polymerase chain reaction in transitional cell tumours of the bladder. *Urol Res* **20**: 297–301

Knowles MA and Williamson M (1993) Mutation of H-ras is infrequent in bladder cancer: confirmation by single-strand conformation polymorphism analysis, designed restriction fragment length polymorphisms, and direct sequencing. *Cancer Res* **53**: 133–139

Knowles MA, Elder PA, Williamson M, Cairns JP, Shaw ME and Law M (1994) Allelotype of human bladder cancer. *Cancer Res* **54**: 531–538

Kong D, Suzuki A, Zou TT, Sakurada A, Kemp LW, Wakatsuki S, Yokoyama T, Yamakawa H, Furukawa T, Sato M, Ohuchi N, Sato S, Yin J, Wang S, Abraham JM, Souza RF, Smolinski KN, Meltzer SJ and Horii A (1997) PTEN1 is frequently mutated in primary endometrial carcinomas [letter]. *Nat Genet* **17**: 143–144

Li J, Yen C, Liaw D, Podsypanina K, Bose S, Wang SI, Puc J, Miliaresis C, Rodgers L, McCombie R, Bigner SH, Giovanella BC, Ittmann M, Tycko B, Hibshoosh H, Wigler MH and Parsons R (1997) *PTEN*, a putative protein tyrosine phosphatase gene mutated in human brain, breast, and prostate cancer. *Science* **275**: 1943–1947

Liaw D, Marsh DJ, Li J, Dahia PL, Wang SI, Zheng Z, Bose S, Call KM, Tsou HC, Peacocke M, Eng C and Parsons R (1997) Germline mutations of the PTEN gene in Cowden disease, an inherited breast and thyroid cancer syndrome. *Nat Genet* **16**: 64–67

Marsh DJ, Dahia PL, Zheng Z, Liaw D, Parsons R, Gorlin RJ and Eng C (1997) Germline mutations in *PTEN* are present in Bannayan–Zonana syndrome. *Nat Genet* **16**: 333–334

Nelen MR, van Staveren WC, Peeters EA, Hassel MB, Gorlin RJ, Hamm H, Lindboe CF, Fryns JP, Sijmons RH, Woods DG, Mariman EC, Padberg GW and Kremer H (1997) Germline mutations in the *PTEN*/*MMAC1* gene in patients with Cowden disease. *Hum Mol Genet* **6**: 1383–1387

Pesche S, Latil A, Muzeau F, Cussenot O, Fournier G, Longy M, Eng C and Liderea R (1998) *PTEN*/*MMAC1*/*TEP1* involvement in primary prostate cancers. *Oncogene* **16**: 2879–2883

Polascik TJ, Cairns P, Chang WYH, Schoenberg MP and Sidransky D (1995) Distinct regions of allelic loss on chromosome 4 in human primary bladder carcinoma. *Cancer Res* **55**: 5396–5399

Presti JC, Jr Reuter VE, Galan T, Fair WR and Cordon-Cardo C (1991) Molecular genetic alterations in superficial and locally advanced human bladder cancer. *Cancer Res* **51**: 5405–5409

Proctor AJ, Coombs LM, Cairns JP and Knowles MA (1991) Amplification at chromosome 11q13 in transitional cell tumours of the bladder. *Oncogene* **6**: 789–795

Rasheed BK, Fuller GN, Friedman AH, Bigner DD and Bigner SH (1992) Loss of heterozygosity for 10q loci in human gliomas. *Genes Chromosomes Cancer* **5**: 75–82

Rempel SA, Schwechheimer K, Davis RL, Cavenee WK and Rosenblum ML (1993) Loss of heterozygosity for loci on chromosome 10 is associated with morphologically malignant meningioma progression. *Cancer Res* **53**: 2386–2392

Rhei E, Kang L, Bogomolniy F, Federici MG, Borgen PI and Boyd J (1997) Mutation analysis of the putative tumor suppressor gene *PTEN*/*MMAC1* in primary breast carcinomas. *Cancer Res* **57**: 3657–3659

Risinger JI, Hayes AK, Berchuck A and Barrett JC (1997) *PTEN*/*MMAC1* mutations in endometrial cancers. *Cancer Res* **57**: 4736–4738

Smeets W, Pauwels R, Laarakkers L, Debruyne F and Geraedts J (1987) Chromosomal analysis of bladder cancer. III. Nonrandom alterations. *Cancer Genet Cytogenet* **29**: 29–41

Steck PA, Pershouse MA, Jasser SA, Yung WK, Lin H, Ligon AH, Langford LA, Baumgard ML, Hattier T, Davis T, Frye C, Hu R, Swedlund B, Teng DH and Tavtigian SV (1997) Identification of a candidate tumour suppressor gene, MMAC1, at chromosome 10q23.3 that is mutated in multiple advanced cancers. *Nat Genet* **15**: 356–362

Teng DH, HU R, Lin H, Davis T, Iliev D, Frye C, Swedlund B, Hansen KL, Vinson VL, Gumpper KL, Ellis L, El-Naggar A, Frazier M, Jasser S, Langford LA, Lee J, Mills GB, Pershouse MA, Pollack RE, Tornos C, Troncoso P, Yung WK, Fujii G, Berson A, Bookstein R, Bolen JB, Tautigian SV and Steck PA (1997) *MMAC1*/*PTEN* mutations in primary tumor specimens and tumor cell lines. *Cancer Res* **57**: 5221–5225

UICC (1978) *TNM Classification of Malignant Tumors, Bladder*, pp. 113–117. Union Internationale Contre le Cancer: Geneva

Wang MR, Perissel B, Taillandier J, Kemeny JL, Fonck Y, Lautier A, Benkhalifa M and Malet P (1994) Nonrandom changes of chromosome 10 in bladder cancer. Detection by FISH. *Cancer Genet Cytogenet* **73**: 8–10

Whang YE, Wu X, Suzuki H, Reiter RE, Tran C, Vessella RL, Said JW, Isaacs WB and Sawyers CL (1998) Inactivation of the tumor suppressor PTEN/MMAC1 in advanced human prostate cancer through loss of expression. *Proc Natl Acad Sci US A* **95**: 5246–5250

 *© Cancer Research Campaign 1999*
